# Management of intra-thoracic anastomotic leakages after esophagectomy: updated systematic review and meta-analysis of endoscopic vacuum therapy versus stenting

**DOI:** 10.1186/s12893-022-01764-z

**Published:** 2022-08-11

**Authors:** Pasquale Scognamiglio, Matthias Reeh, Nathaniel Melling, Marcus Kantowski, Ann-Kathrin Eichelmann, Seung-Hun Chon, Nader El-Sourani, Gerhard Schön, Alexandra Höller, Jakob R. Izbicki, Michael Tachezy

**Affiliations:** 1 Department of General, Visceral and Thoracic Surgery, University Medical Center Hamburg-Eppendorf, Martinistraße 52, 20246 Hamburg, Germany; 2 Department of Interdisciplinary Endoscopy, University Medical Center Hamburg-Eppendorf, Hamburg, Germany; 3 General, Visceral and Transplantation Surgery, University Hospital Münster, Münster, Germany; 4 Department of General, Visceral and Cancer Surgery, University Hospital Cologne, Cologne, Germany; 5 Department for General and Visceral Surgery, University Hospital, Klinikum Oldenburg AöR, Oldenburg, Germany; 6 Institute of Medical Biometry and Epidemiology, University Medical Center Hamburg-Eppendorf, Hamburg, Germany

**Keywords:** Vacuum therapy, Endoscopic stenting, Anastomotic leakage, Esophagectomy

## Abstract

Despite a significant decrease of surgery-related mortality and morbidity, anastomotic leakage still occurs in a significant number of patients after esophagectomy. The two main endoscopic treatments in case of anastomotic leakage are self-expanding metal stents (SEMS) and the endoscopic vacuum therapy (EVT). It is still under debate, if one method is superior to the other. Therefore, we performed a systematic review and meta-analysis of the existing literature to compare the effectiveness and the related morbidity of SEMS and EVT in the treatment of esophageal leakage. We systematically searched for studies comparing SEMS and EVT to treat anastomotic leak after esophageal surgery. Predefined endpoints including outcome, treatment success, endoscopy, treatment duration, re-operation rate, intensive care and hospitalization time, stricture rate, morbidity and mortality were assessed and included in the meta-analysis. Seven retrospective studies including 338 patients matched the inclusion criteria. Compared to stenting, EVT was significantly associated with higher healing (OR 2.47, 95% CI [1.30 to 4.73]), higher number of endoscopic changes (pooled median difference of 3.57 (95% CI [2.24 to 4.90]), shorter duration of treatment (pooled median difference − 11.57 days; 95% CI [− 17.45 to − 5.69]), and stricture rate (OR 0.22, 95% CI [0.08 to 0.62]). Hospitalization and intensive care unit duration, in-hospital mortality rate, rate of major and treatment related complications, of surgical revisions and of esophago-tracheal fistula failed to show significant differences between the two groups. Our analysis indicates a high potential for EVT, but because of the retrospective design of the included studies with potential biases, these results must be interpreted with caution. More robust prospective randomized trials should further investigate the potential of the two procedures.

## Introduction

In the last decades, interdisciplinary treatment and improvement of surgical techniques led to a significant decrease of surgery-related mortality and morbidity after esophageal surgery [1–5]. However, although benchmarking studies showed that a leakage rate of 10–15% is realistic in centers with adequate volume, most studies report rates up to 30% with a high rate of fatal outcomes [6–9].

While cervical anastomotic leakages can usually be managed by wound drainage, intrathoracic and abdominal esophageal leakage requires an individual treatment strategy [9–13]. Undisputed is the need of a surgical revision in case of conduit necrosis [14–16], whereas for contained leakages nonsurgical, radiologic-interventional or endoscopic procedures are more commonly used [14, 16–19]. The two main endoscopic treatments of anastomotic leakage after esophagectomy are the placement of self-expandable metal stents (SEMS), which can still be denoted as the therapeutic gold standard in most parts of the world and, as a more recently introduced method, the endoscopic vacuum therapy (EVT). At least in Germany, the latter has become the more preferred method since its introduction in 2008. The SEMS covers the leak until the defect has healed, with a closure rate of approximately 83% (range 50–100%). Concomitant percutaneous drainages are necessary to control pleural infection and sepsis [20, 21].

EVT is performed by endoscopical positioning of a polyurethane sponge into the esophagus or even into the leak itself. Continuous negative pressure (up to − 125 mmHg) is applied to the sponge through a nasogastric tube. The sponge is regularly changed every 72–96 h [22]. Continuous fluid suction is one of the main advantages of EVT compared to SEMS: it reduces the need for percutaneous or surgical drainage of fluid collections, and thus the complications related to these procedures [23]. In the literature, a closure rate ranging from 66 to 100% has been reported [24, 25]. Other endoscopic interventions, such as endoscopic suture techniques or clipping, have been described only in case series [26, 27].

The aim of this updated systematic review of the currently available literature and meta-analysis is to summarize the growing comparative data regarding the two most frequently used techniques for the treatment of intrathoracic or abdominal anastomotic leakage after esophagectomy, SEMS and EVT, and to increase the existing evidence for these methods [28, 29].

## Methods and data

Search strategy, data collection and data analysis were performed as reported in our previous meta-analysis [28] and are described below.

### Inclusion criteria

We searched for prospective and retrospective studies comparing endoscopic stenting and EVT for the treatment of patients with anastomotic leakage after gastric or esophageal resection and intrathoracic/mediastinal esophago-enteric anastomosis.

### Outcomes

The primary analyzed outcome was the rate of successful leak closure. Secondary outcomes were mortality, ICU and hospitalization time (in days), duration of endoscopic therapy (in days), and number of endoscopic treatments (stent and sponge changes), major and treatment related complications, re-operation rate, stricture and tracheobronchial fistula rate.

### Search strategy

We performed a systematic review according to the guidelines of the Preferred Reporting Items for Systematic Reviews and Meta-analyses (PRISMA) checklist [30], searching for published and unpublished trials without language restrictions using the Cochrane central register of controlled trials (central) and MEDLINE (1 January 2008 to 1 June 2022). Searches were carried out using medical subject headings and free-text words in combination with the search strategy for randomized controlled trials.

The proposed search strategy for MEDLINE (Ovid interface) was: (1) EVT OR vacuum OR sponge. (2) stent OR SEMS. (3) #1 AND #2. The strategy was changed for other databases. In addition, we searched the reference lists of articles retrieved by the search and contacted experts in the field to obtain additional data. We also searched relevant journals and conference abstracts to address the issue of publication bias.

### Data collection and analysis

The titles and abstracts of the manuscripts were independently assessed by two investigators (M.T. and P.S.). Studies that clearly did not meet the inclusion criteria were excluded. The full texts of all possibly relevant articles were evaluated to determine eligibility. Disagreements were resolved by consultation with a third investigator (N.M.)

Independently, the following data were retrieved: authors, year of publication, country, inclusion and exclusion criteria, study methodology, number of treated patients in each group, age and sex of patients, underlying disease, type of resection and reconstruction, neoadjuvant therapy, intervention details, duration of endoscopic treatment, number of endoscopic sessions, risk of bias, and outcomes including endoscopy-related complications, closure rate, re-operation rate, overall hospitalization, and in-hospital mortality. We corresponded with study investigators for further data on methods and results, as required.

### Study quality

Risk of bias was evaluated by the two investigators based on the ROBINS-I score, validating each grade of confounding, selection, classification of and deviation from intervention, missing data, outcome measurement, and selection of reported results [31].

Disagreements were objectively discussed by the two investigators until an agreement was reached.

### Statistical analysis

The treatment effect was measured with odds ratios (ORs) for dichotomous variables and median differences for continuous variables. The homogeneity of effect sizes among studies being pooled was assessed with the I^2^ statistic. A meta‐analysis was conducted regardless of the level of homogeneity. Dichotomous outcomes were pooled using the Mantel–Haenszel method under a random‐effects model. Pooled-effect measures were calculated with 95% confidence intervals (CIs). Estimation of pooled medians was performed using the quantile estimation method [32], with the difference in medians calculated as the median of the EVT group minus the median of the SEMS group.

All statistical analyses were done with the statistical software package R [33] version 4.0.3, the R-packages metafor [34] version 2.4-0, and metamedian [35] version 0.1.5. Sensitivity analyses were planned on the basis of trial quality and the methods of the meta‐analysis, but because of the small number of studies available for meta‐analysis, this was not performed.

## Results

### Description of studies

**Fig. 1:**
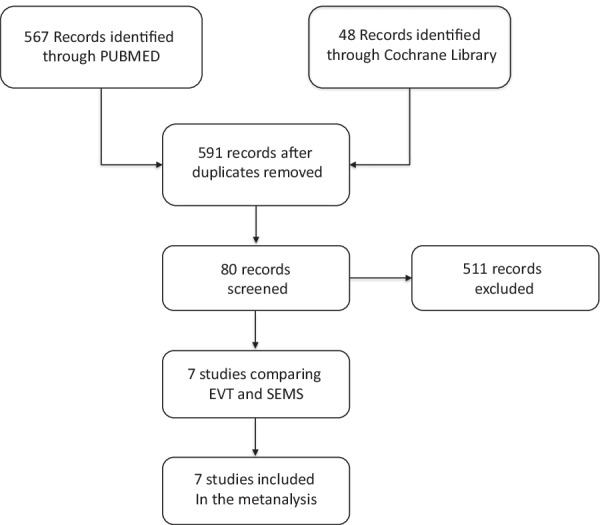
PRISMA flow diagram of study selection

A total of 615 publications were found using the search strategy. After removing 27 duplicate records, we screened the titles and abstracts of 588 records and discarded 508 records, as they did not meet the inclusion criteria. We obtained the full text of 80 articles for in‐depth review and excluded 73, leaving seven retrospective studies to be included in this review [36–42]. The PRISMA flow diagram is provided in Fig. 1.

The studies included 338 patients with esophageal leakage in the analysis (range 18–111). Patient characteristics are summarized in Table 1.

**Table 1:** Patient characteristics

Publication year	Author	Initial treatment method	Country	Patients (n)	Female (n)	(%)	Age (Median (Range))	Oncologic resection (%)	Neoadjuvant therapy (%)	Etiology: surgical anastomic leak (%)	Subtotal esophagectomy (%)	Reconstruction type esophagogastrostomy (%)	Cervical leak	%
2013	Brangewitz	EVT	Germany	32	4	13	63 (45–84)	88	56	94		44	–	–
		SEMS		39	9	23	62 (32–78)	74	15	79		69	–	–
2013	Schniewind	EVT	Germany	17	–	–	–	–	–	100	100	–	3	18
		SEMS		12	–	–	–	–	–	100	100	–	1	8
2015	Mennigen	EVT	Germany	15	1	7	56 (42–76)	100	73	100	100	100	0	0
		SEMS		30	9	30	66 (40–92)	96	43	100	100	100	0	0
2016	Hwang	EVT	South Korea	7	2	29	71 (63–78)	100	–	100	71	–	0	0
		SEMS		11	2	18	67 (55–81)	100	–	100	36	–	1	9
2018	Berlth	EVT	Germany	35	5	14	65 (43–84)	100	53	100	74	74	1	3
		SEMS		76	14	18	64 (43–88)	100	65	100	88	88	0	0
2021	Eichelmann	EVT	Germany	30	3	12	60 (42–78)	100	80	100	100	100	0	0
		SEMS		14	3	23	65 (37–88)	100	62	100	100	100	0	0
2021	El-Sourani	EVT	Germany	13	1	8	60(49–75)	100	10	100	100	100	0	0
		SEMS		7	2	28	56 (49–69)	100	3	100	100	100	0	0

All studies compared EVT with endoscopic stenting; three studies also included surgical revision (Schniewind et al. [40] Eichelmann et al. [41] and El Sourani et al. [42]).

Five studies [36–39, 42] reported the primary outcome of healing rate after endoscopic therapy. By contacting Eichelmann et al. [41] we were also able to obtain the healing rate of their patient cohort. Healing rates from Schniewind et al. [40] were not available for analysis.

Reported secondary outcomes were mortality, ICU and hospitalization time, major and treatment related complications, re-operation rate, stricture rate, esophago-tracheal fistula rate and duration of endoscopic therapy and number of interventions.

### Quality of included studies

We performed ROBINS-I scoring to evaluate the risk of bias in the included retrospective studies [31] (Table 2). The overall risk of bias of the retrospective studies was low to moderate in six studies, but one study showed a serious risk of bias, mainly due to missing data. Because of the small number of included studies, no testing for publication bias for the primary outcome was performed as recommended by the Cochrane Collaboration.

**Table 2:** ROBINS-I Scoring of the selected studies

Study	Study type	Pre-intervention		At intervention	Post-intervention				Overall
		Confounding	Selection	Classification of intervention	Deviation from intervention	Missing data	Measurement of outcomes	Selection of reported results	
Brangewitz et al.	Retrospective	2	2	1	2	1	1	1	2
Schniewind et al.	Retrospective	3	2	1	1	3	3	2	3
Mennigen et al.	Retrospective	1	1	1	1	1	1	1	1
Hwang et al.	Retrospective	1	1	1	1	2	1	2	2
Berlth et al.	Retrospective	1	1	1	1	1	1	1	1
Eichelmann et al.	Retrospective	1	1	1	1	2	1	1	2
El Sourani et al.	Retrospective	2	1	1	1	1	1	1	2

### Outcomes

Patient outcomes are summarized in Table 3. All studies reported results on the duration of hospitalization. The number of endoscopic interventions, treatment duration, successful closure, re-operation rate and in-hospital mortality were reported in six trials, each. Treatment related complications, was reported in five studies. Four studies presented data for major complications and intensive care unit (ICU) duration, three studies for post-therapeutic stricture. However, not all studies could always be included in respective meta-analyses due to different endpoints reported.

**Table 3:** Patient outcome

Year	Author	Initial treatment method	Patients (n)	Median Number of stents/ sponges (range)	Median days treatment duration (range)	Successful closure rate (n)	Treatment related complications (n)	Majpr complications (n)	Esophago- tracheal fistula (n)	Surgical revision (n)	Stricture (n)	Time on ICU days	Median hospitalization in days (range)	In hospital mortality (n)
2013	Brangewitz	EVT	32	7 (5–28)	23 (9–86)	27	8	3	1	1	3	-	48.5 (21–122) (mean und range)	5
		SEMS	39	3 (2–6)	33 (9–132)	21	8	6	0	3	11	-	41 (2–93) (mean und range)	11
2013	Schniewind	EVT	17	–	–	–	–	–	–	0	–	26 ± 19 (mean + SD)	57 ± 30 (mean und SD)	2
		SEMS	12	–	–	–	–	–	–	2	–	38 ± 32 (mean + SD)	62 ± 39 (mean + SD)	5
2015	Mennigen	EVT	15	6.5 (1–18)	26,5 (3–75)	14	0	0	0	0	–	–	58 (23–106)	1
		SEMS	30	1 (1–6)	36 (1–156)	19	1	3	1	4	–	–	53 (13–195)	8
2016	Hwang	EVT	7	4.3 (2–10)	27 (2–84)	7	0	0	–	0	–	–	37.1 (13–128)	–
		SEMS	11	1.6 (1–4)	19.5 (5–21)	7	6	5	–	3	–	–	87.3 (17–366)	–
2018	Berlth	EVT	34	3 (1–9)	12 (3–58)	24	4	–	0	0	1	Median 6 (0–60)	37 (19–118)	3
		SEMS	77	1 (1–3)	27 (1–152)	49	18	–	4	0	5	Median 9 (0–295)	38 (13–296)	11
2021	Eichelmann	EVT	30	6 (1–25)	23 (3–101)	28	–	–	–	–	1	Median 18 (4–107)	47 (14–119)	5
		SEMS	14	1 (1–3)	44 (11–68)	13	–	–	–	–	5	Median 18 (8–38)	34 (17–56)	1
2021	El Sourani	EVT	13	5 (4–18)	24.5 (8–80	12	0	4	–	5	–	Median 38 (9–193)	74 (9–193)	5
		SEMS	7	1 (1–2)	22 (3–31)	6	2	2	–	1	–	Median 20 (16–57)	41 (22–123)	1

**Fig. 2:**
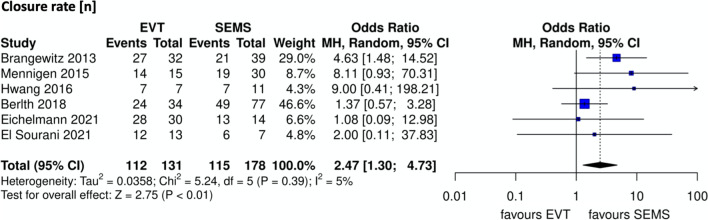
Closure rate

EVT was associated with a significantly higher rate of leak closure compared to stenting (OR 2.47, 95% CI [1.30 to 4.73]) (Fig. 2).

**Fig. 3:**
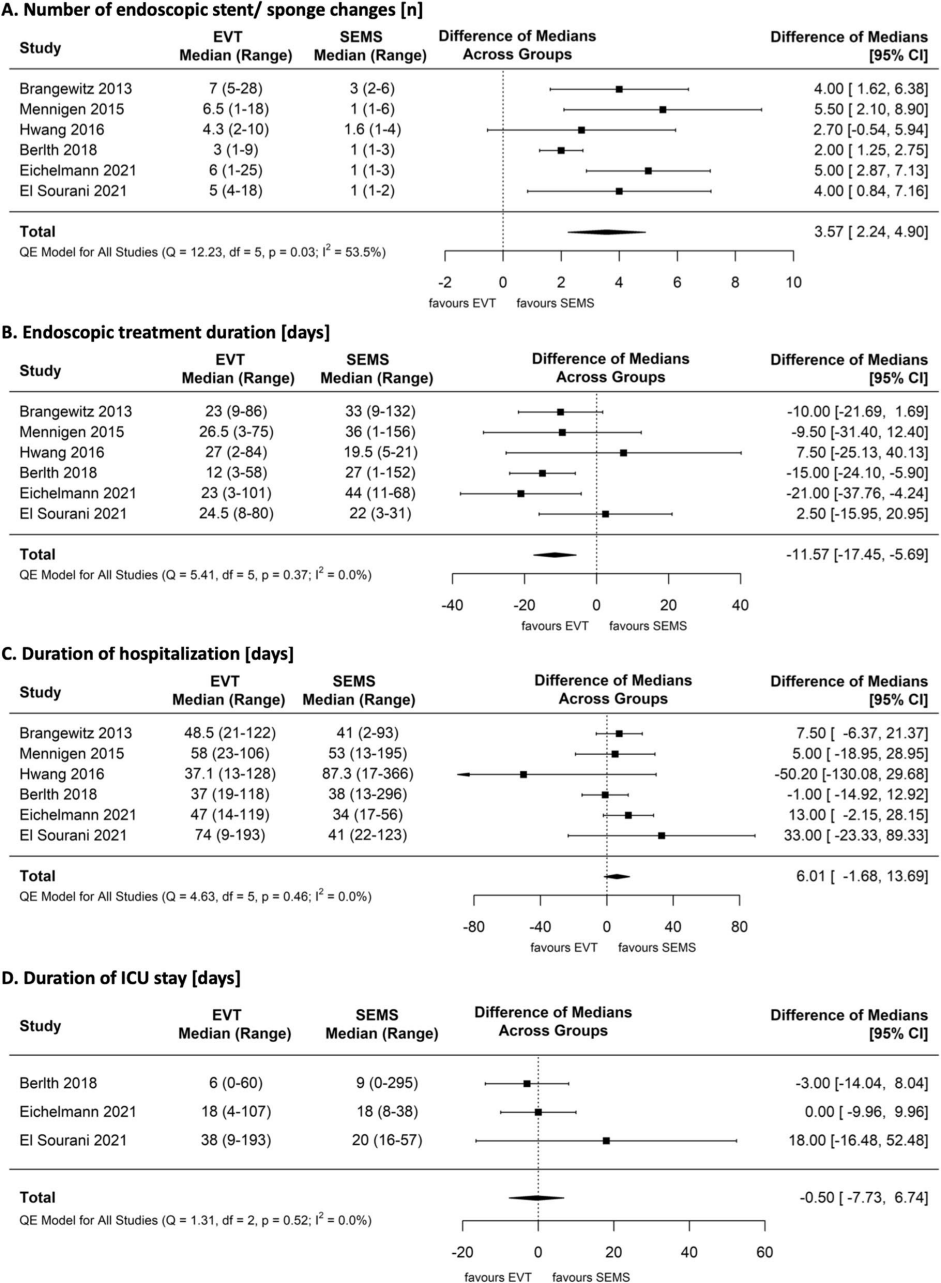
**A** Number of endoscopic stent/sponge changes, **B** endoscopic treatment duration, **C** duration of hospitalization, **D** duration of intensive care unit stay

The number of endoscopic device changes was significantly more frequent in the EVT group than in the SEMS group, with an estimated pooled median difference of 3.57 (95% CI [2.24 to 4.90]) (Fig. 3A). The duration of treatment was generally shorter for patients treated by EVT, with the exception of two studies that showed a shorter, but not significant treatment time for the SEMS group. The estimated pooled median difference (− 11.57 days) was nevertheless statistically significant (95% CI [− 17.45 to − 5.69]) (Fig. 3B).

The duration of overall hospitalization was non superior in one of the groups, with an estimated pooled median difference of 6 days (95% CI [− 1.68 to 13.69]) (Fig. 3C).

Data on the duration of ICU stay could be pooled in three studies, showing no significant differences between the two groups (estimated pooled median difference of − 0.5 days (95% CI [− 7.73 to 6.74]) Fig. 3D).

**Fig. 4:**
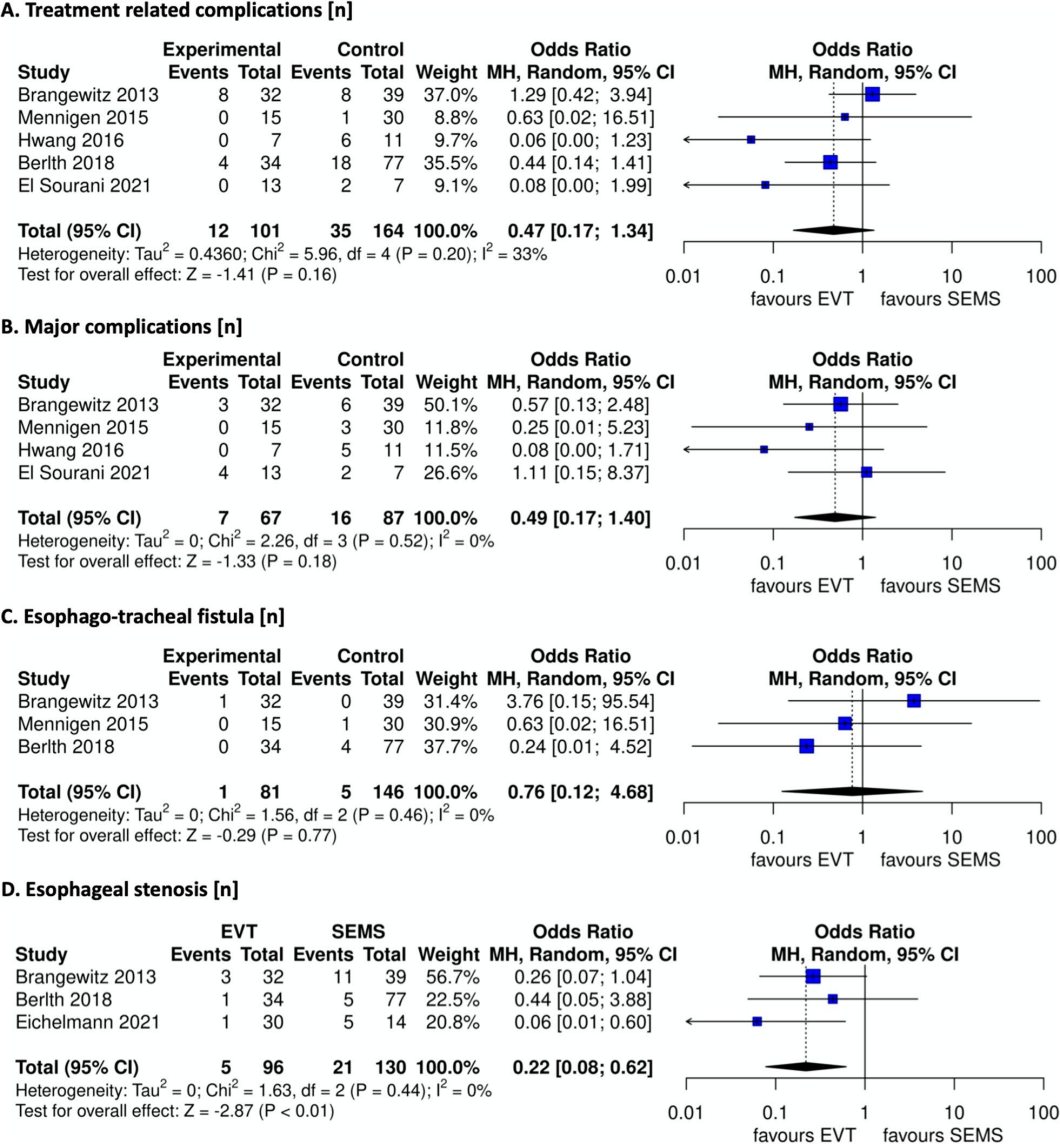
**A** Treatment related complications, **B** major complications, **C** esophago-tracheal fistula, **D** esophageal stenosis/stricture

Treatment related and major complications failed to show significant differences (OR 0.47, 95% CI [0.17 to 1.34]; and OR 0.49, 95% CI [0.17 to 1.40], respectively), even though both were fewer in EVT patients (Fig. 4A, B).

Rate of esophago-tracheal fistula showed no significant differences (OR 0.76, 95% CI [0.12 to 4.68]) (Fig. 4C). Focusing on the stricture rate after healing, it proved to be statistically lower in the EVT group (OR 0.22, 95% CI [0.08 to 0.62]) (Fig. 4D).

**Fig. 5:**
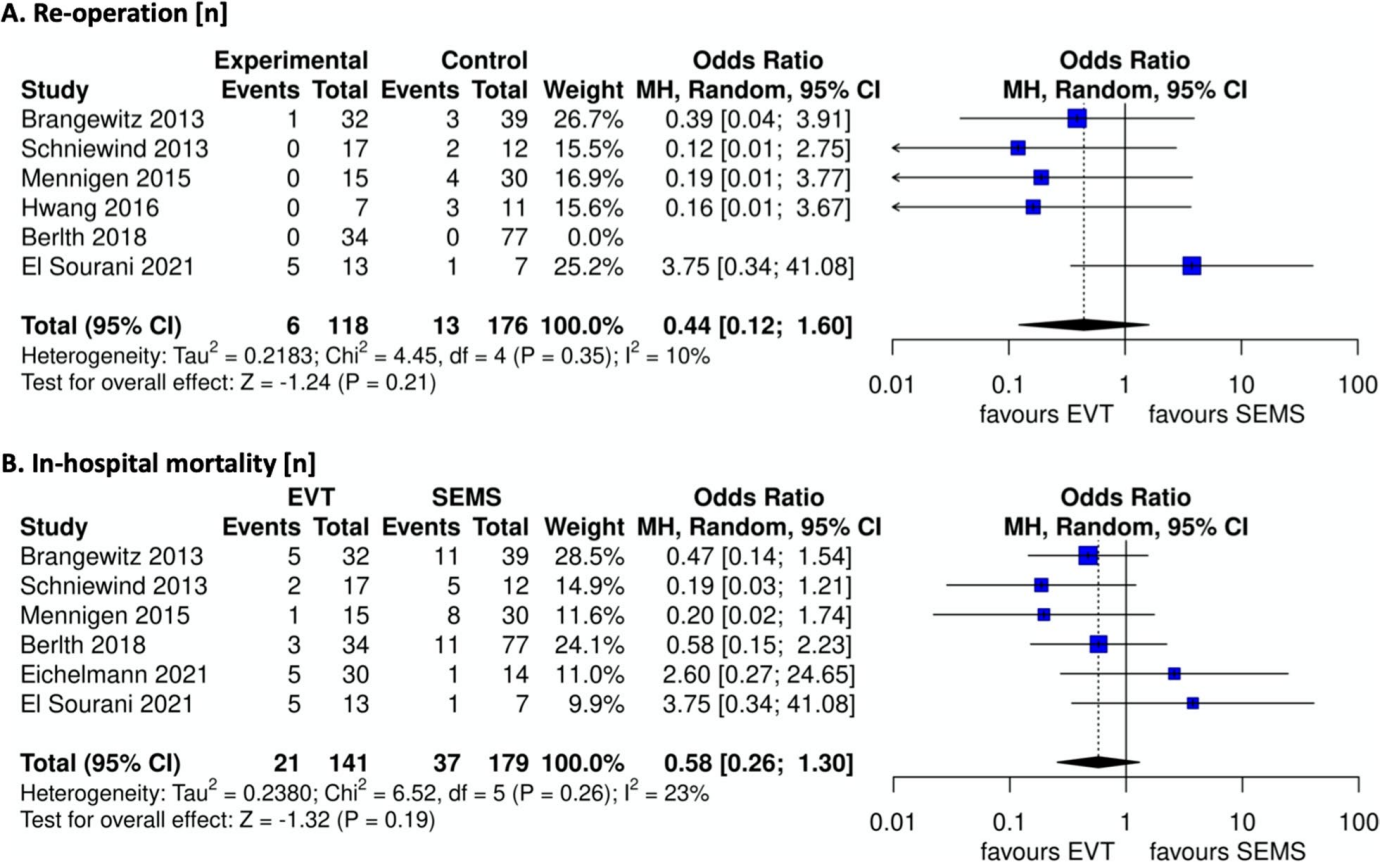
**A** Re-operation, **B** in-hospital mortality

Overall surgical revision rate did not show a significant difference between the two groups (OR 0.44, 95% CI [0.12 to 1.60]). (Fig. 5A). In-hospital mortality failed to show a significantly lower outcome in the group of patients treated with EVT (OR 0.58, 95% CI [0.26 to 1.30]) (Fig. 5B).

Treatment costs were reported only by one study [41]. None of the studies reported data on long-term survival, time to resumption of oral intake or quality of life.

## Discussion

Treatment of anastomotic leakage after esophageal resections remains controversial. A rising number of studies have recently compared the two most frequent techniques, SEMS placement and EVT, but prospective, or even randomized studies are still lacking. In our systematic literature search, we identified seven retrospective comparative studies with a total of 338 patients (range 18–111, median 44 patients) that met our inclusion criteria [36–42]. Compared to earlier published meta-analyses comparing SEMS and EVT, we included two additional studies in the present analysis from 2021 [28, 29, 41–43].

According to the recent meta-analyses, the addition of the two recent studies underlined the significantly better healing rate of EVT compared to placement of SEMS. This primary treatment goal is mainly considered as resolution of leakage-related symptoms and endoscopic or radiologic controls showing closure. Nevertheless, the absence of a clear definition was one of the main problems leading to limited comparability in our analysis.

Despite the finding that most of the studies reported less treatment related complications in the EVT group, the meta-analysis failed to show a significant difference. Moreover, we were able to analyze two complications in detail, the feared esophago-tracheal fistula and the esophageal stenosis after healing of the leakage.

It is hypothesized that the SEMS through erosion or smoldering inflammation and EVT through the negative pressure may provoke a higher esophago-tracheal or -bronchial fistula rate, but our analysis of three studies did not show a significant difference of this feared and often fatal complication [36, 44].

The additional study allowed us to perform a meta-analysis of the stricture rate. This remains one of the factors influencing the most the quality of life of patients after the healing of an anastomotic leakage and showed to be higher in the SEMS group.

Treatment duration was significantly shorter with EVT than with SEMS. However, the result of a shortened healing duration must be evaluated critically, since patients being treated with EVT undergo sponge changes every 3–5 days until healing is reported, whereas stents usually remain in place for 4–6 weeks. Consequently, the exact time of leak closure is unprecise in these cases. Corroborating this hypothesis, ICU and hospitalization time showed no significant differences between the two groups.

This bias may well be reversed by monitoring indirect signs of leakage closure, such as the resolution of the symptoms, the start of oral intake or the absence of further therapeutic interventions. Unfortunately, none of the studies presented these data.

In accordance with our previous findings [28], EVT needed a higher number of endoscopic interventions for changing of the sponge. This allows for better monitoring of local inflammation, endoscopic lavage and debridement, reduction of pleural inflammation and possibly, leakage-associated mortality, on the one hand. On the other hand, this generates more periprocedural stress, and recurrent sedation may also result in complications [45]. In this regard, recent studies suggest the use of a naso-mediastinal drainage system to drain the mediastinal abscess similarly to EVT, eventually reducing the number of endoscopic interventions necessary to change the sponge [46]. Comparative studies with other methods are though still missing.

Another main confounding factor is the heterogeneity of the included patients: in fact, intrinsic characteristics of the leakage, such as the circumferential extent, necessarily affect the outcome. Between the included studies, only Berlth et al. and El Sourani et al. classified the patients based on the leakage grade [36, 42].

Moreover, the SEMS procedure is standardized and reproducible, whereas EVT has many variables which may present differences between institutions: (1) extent of negative pressure, (2) extra- or intraluminal placement of the sponge, (3) time interval between sponge changes, (4) size of the sponge and material.

Specifically, in the included studies, Schniewind et al. used a negative pressure of 70–80 mmHg, with varying intervals between sponge changes [40]. The other studies all reported the same negative pressure of 125 mmHg, and the time between the interventions ranged between 3 and 7 days [36–39, 41]. The goal should be an intracavitary, extra-luminal placement of the polyurethan foam. If the leakage size is too small, either an endoscopic widening of the hole is possible to examine and clean the abscess and to place the sponge inside or alternatively, an intraluminal placement of the sponge is possible [47]. With this, the abscess cavity can be drained, but in our opinion, the cleaning and granulation inducing effect of the EVT is significantly reduced. Aiming to tailor the optimal therapy for each situation, new methods and materials were recently added as a helpful alternative to the classical polyurethan foam, for example other, softer sponge materials (‘white sponge’) or the open pore film drainage. The latter is a great improvement that allows the treatment of smaller leakages and both can be left in situ for longer intervals, due to its limited ingrowth into the granulation tissue [48–50]. Another benefit of the open pore film drainage is the easier handling during the endoscopic placement process and also the removal. Further innovations are made, such as the recently described hybrid SEMS with vacuum therapy or the introduction of more cost-conscious self-made constructions [50, 51]. In our clinical experience, EVT can be applied both in small and large leakages, even with large abscesses and pleural empyema with good results.

In contrast to the recently published meta-analyses, the mortality rate failed to show a significant difference. It must be highlighted, that the recent study of El Sourani and colleagues showed in contrast to the other four included studies an extra-ordinary high rate of reoperations and concomitant mortality, that was described as not related to the leakage or therapy and happened after the endoscopic closure of the leakage [42]. This might highlight the fact, that an early treatment of the anastomotic leakage does not relevantly elevate the mortality rate per se and the majority or mortal complication is based on other concomitant morbidities [52]. However, it would be of special interest, if the leakage related re-operation rate is influenced by the technique of endoscopic therapy and moreover, if the EVT might reduce the rate of surgical thoracic re-interventions, but none of the studies further specified on this.

Importantly, quality of life during the therapy is an aspect that needs paying attention to. The necessity of a permanent nasogastric tube during the whole duration of the EVT is rather discomforting for the patients. In recent case series, Giraldo-Grueso et al. suggested a pharyngostomy as a novel access for EVT. This approach avoids the necessity of the nasogastric tube, allowing for safe transition to outpatient management and reaching results comparable to those of classic EVT. However, data from just six patients were analyzed, so that future studies are needed to confirm the findings [53]. Another technique, that our group is recently working on, is the access to the esophageal leakage via drainage-channels, that can be used for flexible fistuloscopy and percutaneous sponge placement and was recently published for duodenal and rectal leakages [54, 55].

With its increasing usage and already mentioned developments regarding the material and (patient and therapists) comfort, the indications for the method are expanded. Several groups have investigated a potential benefit of EVT placement as a leakage prophylaxis, not only in case of high-risk anastomoses but as a general approach [56, 57]. Moreover, in a recent case series from Loske and colleagues, a double-lumen drain with open-pore film was used with negative pressure to protect the anastomosis from postoperative reflux and allowed an enteral (jejunal) nutrition after esophageal resection, resulting in no anastomotic leakages in all the treated patients [58].

Another significant feature of the two methods is the socio-economic aspect: owing to the higher number of endoscopies performed for EVT and a different cost reimbursement, the deficit of this treatment modality is twice as high as that of SEMS treatment in the German DRG system [59]. Of the analyzed studies, only one other (Eichelmann and colleagues) investigated this aspect. The authors reported, in line with Berlth’s results, higher costs for EVT than for SEMS, with a delta increase of 37%. Nevertheless, they pointed out that the main costs result from the ICU stay, whereas the costs for the two different interventions were comparable, suggesting that the reduction of the length of the ICU stay should be the main aim to reduce overall costs in case of anastomotic leak [41]. However, based on the current available studies, EVT does not show a relevant reduction of the intensive care treatment time, since the two recent studies did not observe a reduction of the ICU stay, contrary to the studies of Schniewind and Berlth which reported shorter ICU stays for EVT patients [36, 40–42].

Because of the risk of bias in the included primary studies, the results of our analysis must be interpreted cautiously. First of all, since all the included studies are retrospective, no blinding of outcome assessment could be performed. Moreover, the lack of information in some of the studies made the risk of bias difficult to assess. Also, since the studies compare medical products, possible commercial interests have to be taken into consideration. Addressing these fundamental aspects, our group recently started a prospective randomized pilot study (RCT), together with the University of Cologne, the ESOLEAK-Trial [60]. We are aware that the data of our meta-analysis indicate a pretty clear picture and one might argue, that a randomized trial is not necessary and moreover, ethical questioning. SEMS is still and undoubted the gold standard in the majority of esophageal cancer centers worldwide. In our point of view, the superiority of the EVT can yet not be proven on a high-level evidence basis due to the aforementioned points, and we will not be able to make a final judgement about the two techniques without a prospective and controlled analysis. With this study, we hope to give a clearer and less biased picture of the efficacy, morbidity and costs of the two therapies and if it indicates a superiority of the EVT, the technique will be further spread in the surgical community.

In conclusion, our meta-analysis confirms that EVT shows a significantly higher success rate and faster healing of esophageal leaks without an elevated treatment related complication rate, but fails to show a clear superiority regarding in-hospital mortality and hospitalization compared to SEMS. Nevertheless, because of the limitations of the analyzed studies, a clear recommendation cannot be made. High quality register studies or even better randomized controlled trials with standardized treatment and outcome parameters are needed to further evaluate these two treatment options.

## Data Availability

All data generated or analysed during this study are included in this published article.
